# Case report: Histoplasmosis presenting as asymptomatic hypercalcemia detected on routine laboratory testing in a pediatric kidney transplant recipient

**DOI:** 10.3389/fped.2022.1058832

**Published:** 2023-01-20

**Authors:** Elizabeth Spiwak, Shrea Goswami, Sara E. Lay, Corina Nailescu

**Affiliations:** ^1^ Department of Pediatrics (Pediatric Nephrology and Hypertension Center), Peyton Manning Children’s Hospital, Indianapolis, IN, United States; ^2^ Department of Pediatrics, Division of Pediatric Nephrology, Indiana University, Indianapolis, IN, United States; ^3^ Department of Radiology, Indiana University, Indianapolis, IN, United States

**Keywords:** histoplasmosis, hypercalcemia, kidney transplant, acute kidney injury (AKI), pulmonary nodules, pediatric

## Abstract

Among all infections occurring in pediatric kidney transplant recipients, approximately 1%–5% are fungal. Most fungal infections occur in the first 6 months following kidney transplantation. We present the case of a 15-year-old boy with a history of a kidney transplant 4 years ago, who was found to have asymptomatic moderate hypercalcemia on routine laboratory testing, along with an acute deterioration of his kidney function markers. The cause of his acute kidney injury was likely related to hypercalcemia. An extensive workup for hypercalcemia revealed infection with *Histoplasma capsulatum* (histoplasmosis) with multiple pulmonary nodules. Hypercalcemia that was initially refractory to medical management resolved after initiating the antifungal treatment. Fungal granulomatous infections such as histoplasmosis should be considered in the differential diagnosis of hypercalcemia in an asymptomatic pediatric kidney transplant recipient.

## Introduction

The goal of immunosuppression in any organ transplantation is to reduce the risk of rejection while simultaneously outweighing the risk of overwhelming infections and malignancies caused by over-immunosuppression (1).

Despite all the advancements in the field of immunosuppression, infection remains the leading cause of patient mortality and graft loss, as well as the leading cause of hospitalizations in pediatric kidney transplant recipients (2). Although fungal infections are relatively rare in transplant recipients, they still comprise about 1%–5% of all infections in these patients and most commonly occur in the first 6 months post-transplant (3, 4). Post-transplantation disseminated histoplasmosis is very rare in the kidney transplant population, even within endemic areas (5). In a large retrospective case series of histoplasmosis in adult solid organ transplant patients, including kidney transplant, almost half of all cases occurred during the first 2 years after transplant and a third occurred during the first year post-transplant (6). We present a case of asymptomatic hypercalcemia 4 years post-transplant secondary to *Histoplasma capsulatum* (histoplasmosis) infection in a pediatric kidney transplant recipient.

## Initial presentation

A 15-year-old boy with a history of end-stage kidney disease (ESKD) secondary to posterior urethral valves and renal dysplasia requiring a kidney transplant presented for hospital admission after an acute kidney injury (AKI). He was at the 8th percentile for both height and weight. Failure to thrive workup revealed no secondary causes or functional delays; hence, the most likely cause was presumed long-standing ESKD. At our institution, gastrostomy tube (GT) placement is routine for children below a certain age (roughly 8–12 years old) or any child who is cognitively delayed. This patient was transplanted at age 11 years and had a GT pretransplantation for poor growth, so it was left in place post-transplantation to assist with water intake goals as he was not willing to drink the desirable goal by mouth. On routine follow-up labs, he was noted to have an elevated creatinine of 1.84 mg/dl (baseline around 1 mg/dl). His mother reported a history of respiratory syncytial virus 2 months prior. Since that infection, his mother felt it was a constant struggle to “keep labs normal.” She attributed this struggle to his inability to maintain the recommended fluid goal of 2.5–3 L/day. A review of the systems revealed that he was fatigued and had a mild headache a few days prior to admission. The mother and patient denied any fever, change in appetite, cough, congestion, rhinorrhea, or shortness of breath. On physical examination, his weight was 44.1 kg, which was slightly decreased from the previous weight of 44.5 kg 1 month prior. He was in no distress, his lungs were clear, and his heart sounds were normal. He had some peeling skin on his face, but otherwise, no rashes were noted. There was no lymphadenopathy. He had a soft and nontender abdomen, and his GT and Monti channel tube sites looked normal, with no erythema or drainage. His immunosuppression regimen included tacrolimus and mycophenolate mofetil but no steroids, as he was on a steroid-sparing protocol. He had no history of rejection since his transplantation. A repeat lab sample on day 2 of hospitalization showed sodium 136 mmol/L, chloride 103 mmol/L, BUN 38 mg/dl, Cr 1.66 mg/dl, potassium 4.8 mmol/L, bicarbonate 23 mmol/L, glucose 106 mg/dl, calcium moderately elevated at 13.0 mg/dl, and elevated phosphorus at 6.1 mg/dl. Albumin was on the low end of normal at 3.4 g/dl, and ionized calcium was high at 1.53 mmol/L. His lactate dehydrogenase (LDH) was also elevated at 326 U/L. His urinalysis showed pyuria with 10–20 WBCs/hpf noted on microscopic urinalysis; however, this was his baseline, thought to be due to microbial contamination of his abnormal urinary tract, as he had been following a chronic intermittent catheterization regimen.

In addition, he had intermittent, unexplained lymphopenia about 6 months prior to this hospitalization and counts were down right around the time of this admission. His lymphocyte count ranged from 500 to 1,600/µl.

## Initial hospital course and diagnostic assessment of AKI (days 1–4)

**Figure 1:**
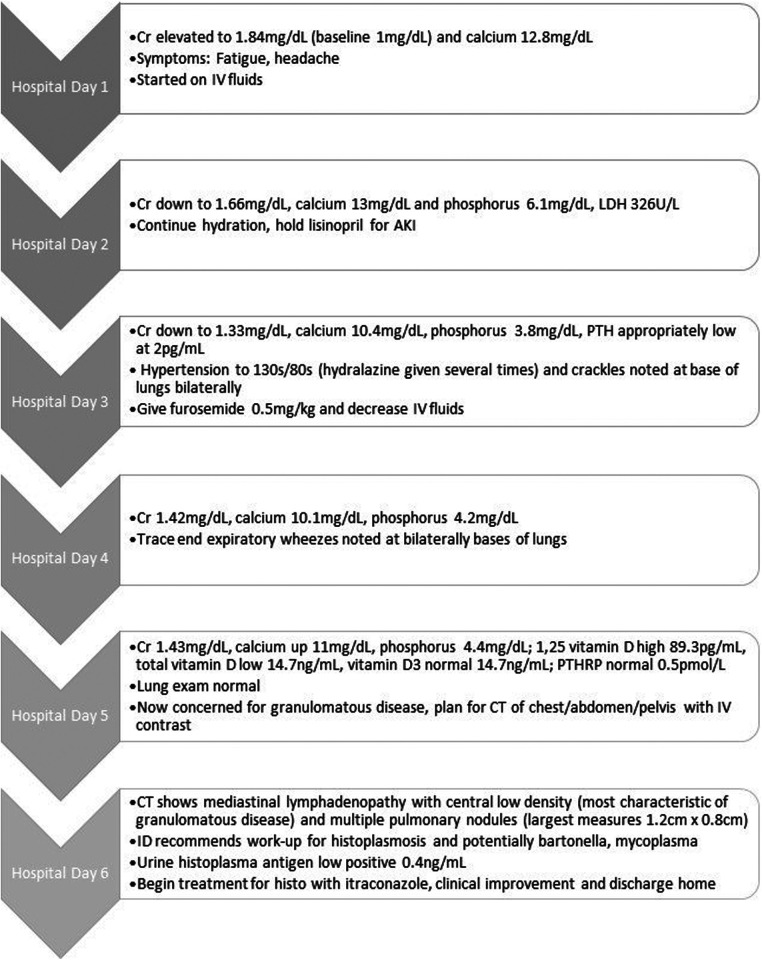
Timeline of hospital course with significant findings each day.

Upon admission, his AKI workup included imaging of the allograft, infectious workup (cytomegalovirus, Epstein–Barr virus, polyoma BK virus testing), and fluid management. He was given 1 L of bolus and placed on maintenance IV fluids calculated for weight. There was initial difficulty in obtaining labs the first night of admission, but they were obtained the following morning. On day 2 of hospitalization, he was noted to have elevated blood pressure and developed crackles at the lung bases bilaterally, which was attributed to fluid overload. His weight had also increased from 44 to 48 kg (9%). To manage the fluid overload and hypercalcemia, furosemide was given on day 3 of hospitalization. Since the creatinine and calcium initially improved with hydration and furosemide, it was assumed that the etiology of the AKI was a combination of prerenal (dehydration) and intrinsic (related to hypercalcemia) (see Figure 1).

## Later hospital course and diagnostic assessment of hypercalcemia (days 5 and 6)

**Figure 2:**
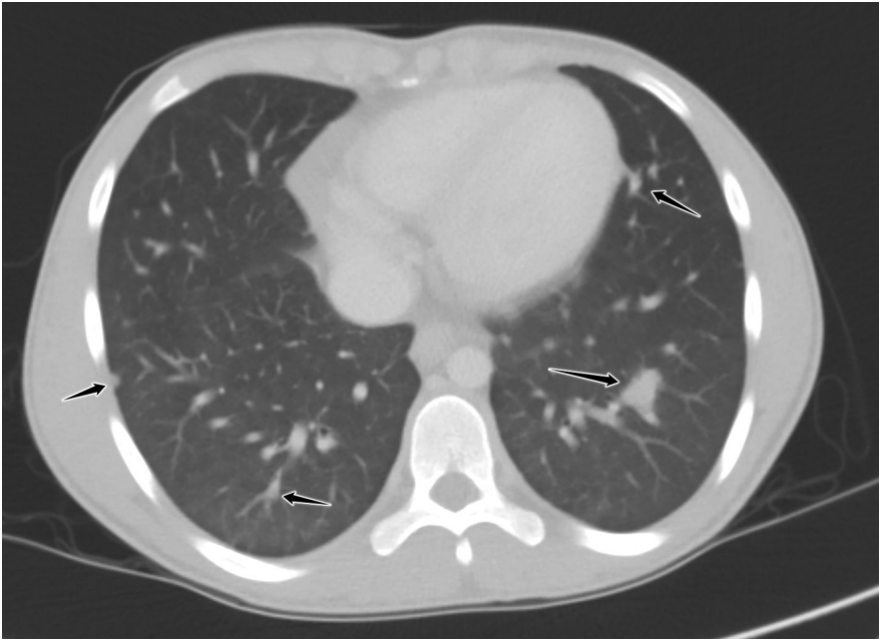
Axial reformatted, lung window CT image through the lower chest demonstrating multiple noncalcified, well-demarcated pulmonary nodules throughout all lobes (black arrows).

**Figure 3:**
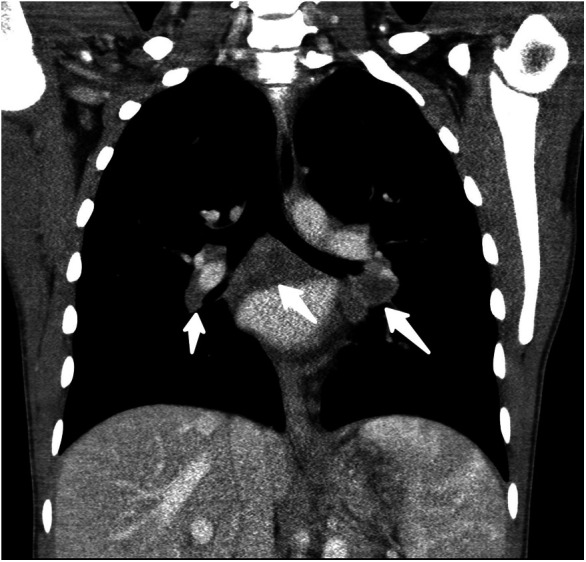
Coronal reformatted, soft-tissue window, CT image through the mediastinum with low attenuation, noncalcified, bihilar, and subcarinal adenopathy (white arrows).

**Figure 4:**
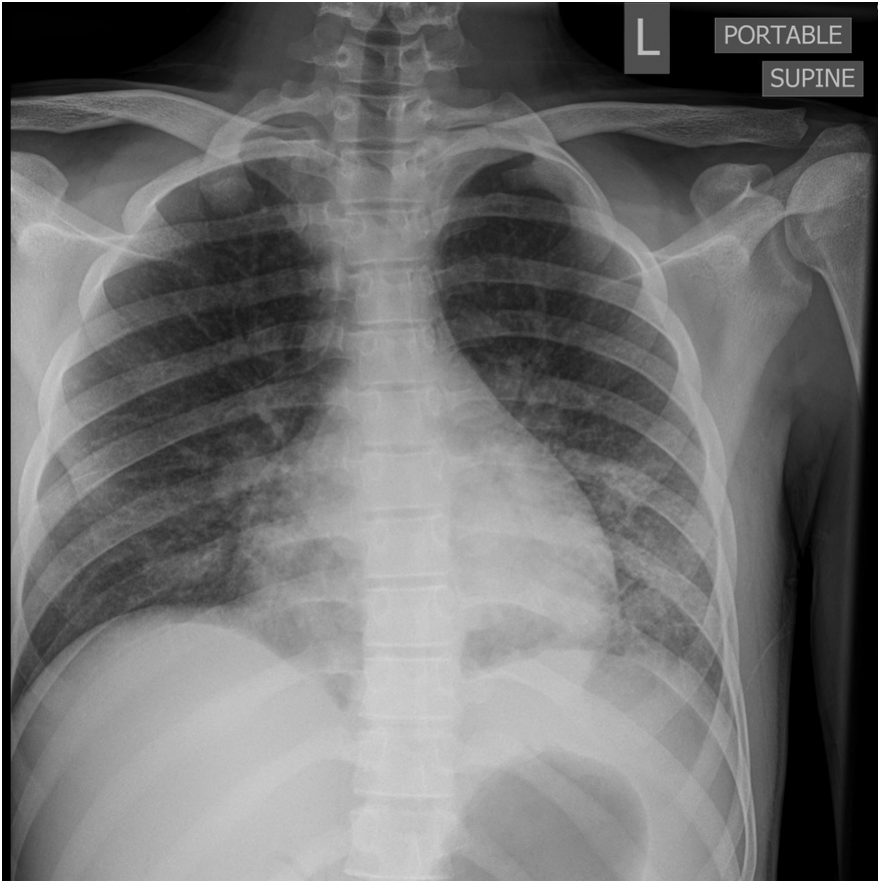
AP portable chest radiograph with diffuse but lower lobe predominant micronodular opacities and air bronchograms.

By day 5 of hospitalization, calcium was again elevated at 11 mg/dl. Upon evaluation of hypercalcemia, labs from day 5 showed 1,25 vitamin D level 89.3 pg/ml (normal 19.9–79.3 pg/ml), vitamin D total level 14.7 ng/ml (normal 30–100 ng/ml), vitamin D3 level at 14.7 ng/ml, and parathyroid hormone (PTH) 2 pg/ml. The differential diagnosis for hypercalcemia with appropriately suppressed PTH and high 1,25 vitamin D includes vitamin A toxicity, hyperthyroidism, lymphoma, or granulomatous diseases. Vitamin A level was normal at 51.6 mcg/dl, and thyroid studies were normal [thyroid stimulating hormone (TSH) 0.728 mcU/ml and T4 1.2 ng/dl], so the focus turned to granulomatous disease. Computed tomography (CT) revealed mediastinal lymphadenopathy that was granulomatous in nature and pulmonary nodules (see Figures 2, 3). He had a chest radiograph done 2 months prior to admission, which was compared to this CT. Radiology noted that, in hindsight, there may have been findings consistent with histoplasmosis, specifically diffuse micronodular and hazy pulmonary opacities with bilateral hilar fullness (see Figure 4). Infectious disease service was consulted, and a purified protein derivative (PPD skin test) and histoplasmosis testing were recommended. Tuberculosis was unlikely as he lacked the standard risk factors, and PPD was negative. Sarcoidosis was also unlikely as he did not meet the typical profile (he was white race, male sex, and young age). With these two causes of granulomatous diseases essentially ruled out, the most likely diagnosis was histoplasmosis. Urine histoplasma antigen was low positive at 0.4 ng/ml, and serum IgG and IgM antibodies were sent. Given the high suspicion for histoplasmosis (based on the clinical picture and endemic region of histoplasmosis in our state) (7) and immunocompromised state, infectious disease recommended initiating treatment with 200 mg daily of itraconazole to complete a 12-month course. Itraconazole was initiated on day 14 of hospital admission with a subsequent decrease in serum calcium to below 10.5 mg/dl, which remained in the normal range (9.3–9.8 mg/dl) after discharge.

Ultimately, his serum histoplasmosis antigen resulted positive at 1.32 ng/ml (any detection is marked as positive with a detectable range of 0.4–19 µl), and the diagnosis of histoplasmosis was confirmed as the etiology for hypercalcemia, which had caused the AKI. One month after diagnosis, his only complaints were alopecia as a side effect of itraconazole. His creatinine returned to his baseline at 1.18 mg/dl. He did not experience any relapse of his histoplasmosis in his most recent visit in 2022 (about 5 years after the admission and diagnosis), and his creatinine continues to remain at his baseline of 1.15 mg/dl. He did not have any further imaging of his chest. There were no repeat vitamin D levels done.

Interestingly, his lymphocyte counts have been in the normal range since this histoplasmosis infection and admission.

## Discussion

Hypercalcemia resulting in AKI, especially when refractor to fluid resuscitation and calcium-excreting diuretics, necessitates a thoughtful investigation into the possible etiologies to guide targeted therapy (8, 9). In our case, the patient did not fully respond to hydration and calcium-excreting diuretics; therefore, a further thorough evaluation for causes of hypercalcemia was implemented.

Hypercalcemia can be caused by granulomatous diseases, including infections like histoplasmosis (10). This has been noted in a case series done on adult-age kidney transplant patients to be unresponsive to parathyroid hormone influence and can be refractory to treatment (11). In our case, PTH was appropriately low as a response to hypercalcemia but refractory to standard treatment of hydration. Indeed, granulomatous diseases have been well described in solid organ transplant recipients, including kidney transplants (12). However, the presentation is usually different, including respiratory symptoms (13) and weight loss (14). One unique aspect of our case was related to the patient's age, as, to our knowledge, histoplasmosis in pediatric kidney transplant recipients is rare and only a few case reports have been described (15–18). While our patient presented exclusively with hypercalcemia, other pediatric cases in the literature have had significant clinically apparent symptoms.

In the United States, the highest incidence of infections with *Histoplasma capsulatum*, known as histoplasmosis, occurs in a region often referred to as the “Histo Belt,” where up to 90% of the adult population has been at some point infected by histoplasmosis. This region includes the entire states of Arkansas, Kentucky, Missouri, Tennessee, and West Virginia, as well as large portions of Alabama, Illinois, Indiana, Iowa, Kansas, Louisiana, Maryland, Mississippi, Nebraska, Ohio, Oklahoma, Texas, and Virginia (19) Therefore, transplant clinicians working in medical centers within the “Histo Belt,” such as our center, need to have a higher index of suspicion for histoplasmosis.

One limitation we experienced in the care of this patient was a delay due to the send-out processing of some laboratory tests. Given that many transplant patients improve with fluids alone, his initial treatment of intravenous fluids was appropriate and resulted in an initial improvement in AKI and hypercalcemia. Furosemide provided some improvement in the hypercalcemia, but this was not sustained. As the etiology of hypercalcemia was due to an infection, the resolution of hypercalcemia would likely not have occurred without antifungal therapy, which was started several days into his course.

In conclusion, histoplasmosis should be considered in the differential diagnosis of hypercalcemia in a pediatric kidney transplant recipient that does not promptly and completely resolve with typical treatment such as intravenous hydration and calcium-excreting diuretics, even when asymptomatic, particularly if the patient lives in one of the high incidence areas.

## Data Availability

The original contributions presented in the study are included in the article/Supplementary Material, further inquiries can be directed to the corresponding author.
